# The Validity of SOFA Score to Predict Mortality in Adult Patients with Cardiogenic Shock on Venoarterial Extracorporeal Membrane Oxygenation

**DOI:** 10.1155/2020/3129864

**Published:** 2020-09-08

**Authors:** Mohamed Laimoud, Mosleh Alanazi

**Affiliations:** ^1^Adult Cardiac Surgical Intensive Care Unit (CSICU), King Faisal Specialist Hospital & Research Center, Riyadh, Saudi Arabia; ^2^Critical Care Medicine Department, Cairo University, Cairo, Egypt

## Abstract

**Background:**

Venoarterial ECMO is increasingly used in resuscitation of adult patients with cardiogenic shock with variable mortality reports worldwide. Our objectives were to study the variables associated with hospital mortality in adult patients supported with VA-ECMO and to determine the validity of repeated assessments of those patients by the Sequential Organ Failure Assessment (SOFA) score for prediction of hospital mortality. We retrospectively studied adult patients admitted to the cardiac surgical critical care unit with cardiogenic shock supported with VA-ECMO from January 2015 to August 2019 in our tertiary care hospital.

**Results:**

One hundred and six patients supported with VA-ECMO were included in our study with in-hospital mortality of 56.6%. The mean age of studied patients was 40.2 ± 14.4 years, and the patients were mostly males (69.8%) with a mean BMI of 26.5 ± 7 without statistically significant differences between survivors and nonsurvivors. Presence of CKD, chronic atrial fibrillation, and cardiac surgeries was significantly more frequent in the nonsurvivors group. The nonsurvivors had more frequent AKI (*p* < 0.001), more haemodialysis use (*p* < 0.001), more gastrointestinal bleeding (*p* = 0.039), more ICH (*p* = 0.006), and fewer ICU days (*p* = 0.002) compared to the survivors group. The mean peak blood lactate level was 11 ± 3 vs 16.7 ± 3.3, *p* < 0.001, and the mean lactate level after 24 hours of ECMO initiation was 2.2 ± 0.9 vs 7.9 ± 5.7, *p* < 0.001, in the survivors and nonsurvivors, respectively. Initial SOFA score ≥13 measured upon ICU admission had a 85% sensitivity and 73.9% specificity for predicting hospital mortality [AUROC = 0.862, 95% CI: 0.791–0.932; *p* < 0.001] with 81% PPV, 79.1% NPV, and 80.2% accuracy while SOFA score ≥13 at day 3 had 100% sensitivity and 91.3% specificity for predicting mortality with 93.8% PPV, 100% NPV, and 96.2% accuracy [AUROC = 0.995, 95% CI: 0.986–1; *p* < 0.001]. The ∆1 SOFA (3-1) ≥2 had 95% sensitivity and 93.5% specificity for predicting hospital mortality [AUROC = 0.958, 95% CI: 0.913–1; *p* < 0.001] with 95% PPV, 93.5% NPV, and 94.3% accuracy. SOFA score ≥15 at day 5 had 98% sensitivity and 100% specificity for predicting mortality with 99% accuracy [AUROC = 0.994, 95% CI: 0.982–1; *p* < 0.001]. The ∆2 SOFA (5-1) ≥2 had 90% sensitivity and 97.8% specificity for predicting hospital mortality [AUROC = 0.958, 95% CI: 0.909–1; *p* < 0.001] with 97.8% PPV, 90% NPV, and 94.8% accuracy. Multivariable regression analysis revealed that increasing ∆1 SOFA score (OR = 2.506, 95% CI: 1.681–3.735, *p* < 0.001) and increasing blood lactate level (OR = 1.388, 95% CI: 1.015–1.898, *p* = 0.04) were significantly associated with hospital mortality after VA-ECMO support for adults with cardiogenic shock.

**Conclusion:**

The use of VA-ECMO in adult patients with cardiogenic shock is still associated with high mortality. Serial evaluation of those patients with SOFA score during the first few days of ECMO support is a good predictor of hospital mortality. Increase in SOFA score after 48 hours and hyperlactataemia are significantly associated with increased hospital mortality.

## 1. Background

The reported mortality among adult patients with cardiogenic shock supported with VA-ECMO varies widely due to differences in patients background and clinical conditions [1–8]. The patients who undergo cardiac surgery may experience refractory postcardiotomy cardiogenic shock associated with increased morbidity and mortality. In these patients, VA-ECMO is considered as a rescue measure to achieve temporary circulatory and respiratory support allowing recovery of cardiac functions or bridging to further therapeutic options [9–14]. We conducted this study to detect the variables associated with hospital mortality in adult patients supported with VA-ECMO and to determine the validity of repeated assessments of those patients by SOFA score for prediction of hospital mortality.

## 2. Methods

### 2.1. Data Source

The study was approved by our institute ethics committee board, and the requirement for informed patient consent was waived because of the retrospective nature of the study. The Hospital Integrated Compliance Information System (ICIS) provided the database of our studied patients. The database included demographic, clinical, laboratory, and operative data, ICU and wards daily records, devices inserted, bedside procedures, and hospital discharge of all patients.

### 2.2. Patients Selection

We retrospectively evaluated consecutive patients who received VA-ECMO support between January 2015 and August 2019 at our tertiary care hospital. We enrolled patients ≥18 years old in the study if they received VA-ECMO support for refractory cardiogenic shock. Exclusion criteria for patient selection were an age <18 years and veno-venous ECMO support.

### 2.3. ECMO Initiation and Patients Management

Venoarterial ECMO support was initiated by venous drainage from the femoral vein or right atrium with extracorporeal oxygen exchange and then return to the arterial system via the femoral artery (peripheral ECMO) or ascending aorta (central ECMO). In case of peripheral ECMO, an additional 6 Fr catheter was inserted distally into the femoral artery to avoid significant leg ischemia.

ECMO initiation and management were performed by trained ECMO team members. Daily checking of the ECMO oxygenator membrane and circuits was done by experienced perfusionists. After ECMO initiation, blood flow was adjusted based on clinical assessments including clearance of hyperlactatemia, mixed venous oxygen saturation, normalization of mean arterial blood pressure and urine output.

Checking of complete blood count and coagulation profile was routinely done for all patients before ECMO support. At ECMO initiation, a heparin bolus (intravenous 80 units/kg) was given, and then, continuous infusion with unfractionated heparin was maintained. The heparin bolus was not given if activated clotting time (ACT) was more than 300 seconds; then, serial measurements every hour were done till ACT was below 300 seconds and heparin infusion started. The heparin dose was adjusted according to the activated partial thromboplastin time (targeting 1.5–2-fold the normal-control value), AT III (goal 80–120%), heparin assay (target 0.3–0.7 units/ml), and clinical tolerance. Cryoprecipitate transfusions were used to keep fibrinogen more than 1 gm/L, and platelets transfusions were used to keep count more than 50 (10^9^/L).

### 2.4. The Studied Variables

Demographic, clinical, and laboratory data of studied patients were collected. The SOFA score was calculated on admission and then every 48 hours for 2 times. The worst values for each variable in the 24 hours period were used during score calculation. The assumed Glasgow Coma Scale (GCS) was used to assess the neurological status in studied patients. As part of our ICU policy, daily sedation vacation was done to assess the neurological state of patients including GCS assessment, any signs of lateralization, and brain stem reflexes. Urgent brain imaging with CT scanning was done within few hours in cases of delayed awakening or signs of lateralization. Regarding the coagulation component of SOFA score, we used the lowest platelet count before transfusions (if given). For the patients who developed renal impairment necessitating renal replacement therapy, we gave them a score of 4 for the renal component of SOFA score (Table 1).

The Δ SOFA score was the difference between 2 subsequent scores. The Δ1 SOFA score was the difference between SOFA score at day 3 and the admission score. The Δ2 SOFA score was the difference between SOFA score at day 5 and the admission scores [15–17].

### 2.5. Statistical Analysis

The patient characteristics were summarized using mean (±standard deviation) or median (interquartile range (IQR)) for continuous variables and as frequency and percentage for categorical variables. Normality of data was checked using the Kolmogorov–Smirnov normality test. Comparisons between numerical data were done using Student's *t*-test or Mann–Whitney accordingly. Ordinal data were compared using the chi square test. *p* values less than 0.05 were considered statistically significant. Assessment of the areas under the receiver operating characteristics (ROC) curves was performed. Logistic regression analysis was done to get the odds ratios (OR) and 95% confidence intervals with hospital mortality as the dependent variable. Graphs were used to illustrate some information. Statistical tests were done using the Statistical Package of Social Science Software program, version 23 (SPSS).

## 3. Results

### 3.1. Baseline and Clinical Data

One hundred and six patients supported with VA-ECMO were included in our study after exclusion of pediatric cases and patients with VV-ECMO. The in-hospital mortality was 56.6%. The mean age of studied patients was 40.2 ± 14.4 years, and the patients were mostly males (69.8%) with a mean BMI of 26.5 ± 7 without statistically significant differences between survivors and nonsurvivors. Presence of chronic kidney disease was significantly more frequent in the nonsurvivors group (*p*=0.001). Chronic atrial fibrillation (*p*=0.006) and oral anticoagulation (*p*=0.005) were statistically more frequent in the nonsurvivors group. There were no statistically significant differences between the survivors and nonsurvivors regarding underlying heart disease, diabetes mellitus, systemic hypertension, left ventricle EF, nor use of IABP. Cardiac surgeries were significantly more frequent in the nonsurvivors group but without significant differences regarding cardiopulmonary bypass or aortic cross clamping times. Central VA-ECMO was more frequent in the nonsurvivors, while peripheral VA-ECMO was more frequent in the survivors group (*p*=0.006) without significant differences regarding ECMO days (*p*=0.21). The nonsurvivors had more frequent AKI (*p* ≤ 0.001), more haemodialysis use (*p* ≤ 0.001), more GI bleeding (*p*=0.039), more ICH (*p*=0.006), and fewer ICU days (*p*=0.002) as compared to the survivors group (Table 2).

### 3.2. Laboratory Data of Studied Patients

At ECMO initiation, the mean blood lactate level was 4.4 ± 1.5 vs 7.2 ± 2, *p* < 0.001; the mean base excess was −7.3 ± 3.6 vs −10.4 ± 3.1, *p* < 0.001, in the survivors and nonsurvivors, respectively. As compared to the survivors, the nonsurvivors had higher serum creatinine level (*p* = 0.018) without significant other laboratory differences (Table 3).

During ECMO support, the mean peak blood lactate level was 11 ± 3 vs 16.7 ± 3.3, *p* < 0.001, and the mean lactate level after 24 hours of ECMO initiation was 2.2 ± 0.9 vs 7.9 ± 5.7, *p* < 0.001, in the survivors and nonsurvivors, respectively. As compared to the survivors, the nonsurvivors had lower serum albumin level (*p* = 0.01) and higher serum creatinine (*p* = 0.017) and bilirubin levels (*p* = 0.03). The nonsurvivors had significant thrombocytopenia (*p* < 0.001) and lower aPTT and PTT ratio compared to the survivors (Table 4).

### 3.3. SOFA Scoring of Patients

**Figure 1:**
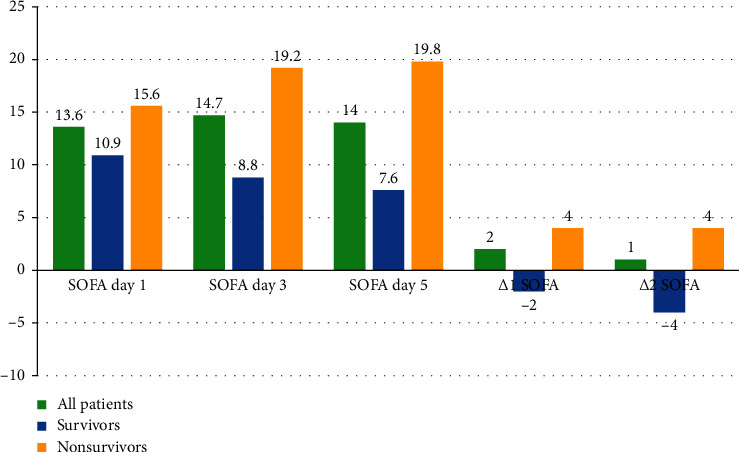
SOFA scoring of studied VA-ECMO patients.

The mean SOFA score at day 1 was 10.9 ± 2.8 vs 15.6 ± 2.9, *p* < 0.001, the mean SOFA score at day 3 was 8.8 ± 2.6 vs 19.2 ± 2.5, *p* < 0.001, and the mean SOFA score at day 5 was 7.6 ± 2.2 vs 19.8 ± 2.5, *p* < 0.001, in the survivors and nonsurvivors, respectively. The median ∆1 SOFA (day 3-1) was −2 (−3 to −1) vs 4 (3–5), *p* < 0.001, and the median ∆2 SOFA (day 5-1) was −4 (−5 to −2) vs 4 (3–6), *p* < 0.001, in the survivors and nonsurvivors, respectively (Table 5 and Figure 1).

**Figure 2:**
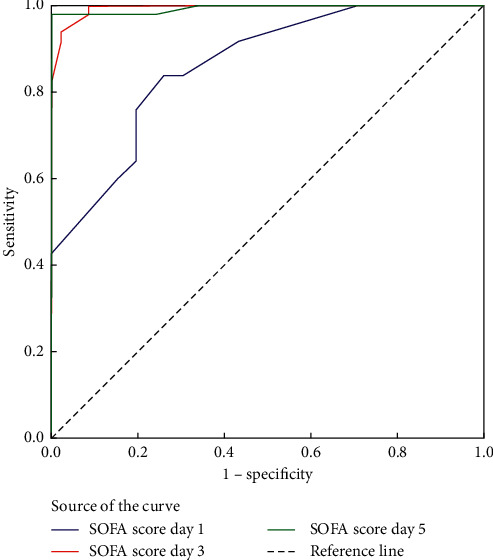
ROC of SOFA scores in differentiating mortality.

**Figure 3:**
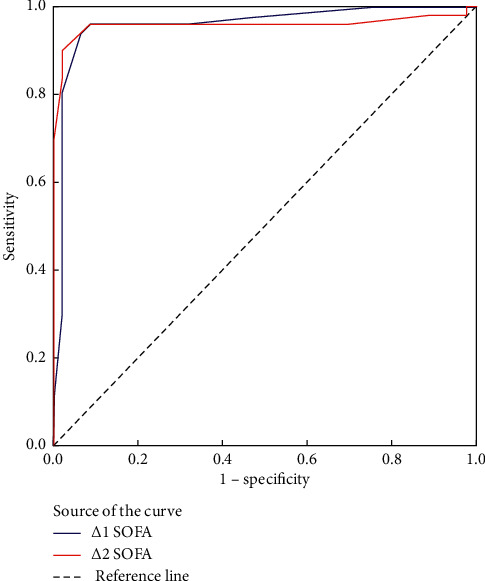
ROC of ∆1 SOFA and ∆2 SOFA in differentiating mortality.

Initial SOFA score ≥13 measured upon ICU admission had 85% sensitivity and 73.9% specificity for predicting hospital mortality [AUROC curve = 0.862, 95% CI: 0.791–0.932; *p* < 0.001] with 81% PPV, 79.1% NPV, and 80.2% accuracy while SOFA score ≥13 at day 3 had 100% sensitivity and 91.3% specificity for predicting mortality with 93.8% PPV, 100% NPV, and 96.2% accuracy [AUROC = 0.995, 95% CI: 0.986–1; *p* < 0.001]. The ∆1 SOFA (3-1) ≥2 had 95% sensitivity and 93.5% specificity for predicting hospital mortality [AUROC = 0.958, 95% CI: 0.913–1; *p* < 0.001] with 95% PPV, 93.5% NPV, and 94.3% accuracy. SOFA score ≥15 at day 5 had 98% sensitivity and 100% specificity for predicting mortality with 99% accuracy [AUROC = 0.994, 95% CI: 0.982–1; *p* < 0.001]. The ∆2 SOFA (5-1) ≥2 had 90% sensitivity and 97.8% specificity for predicting hospital mortality [AUROC = 0.958, 95% CI: 0.909–1; *p* < 0.001] with 97.8% PPV, 90% NPV, and 94.8% accuracy (Table 6; Figures 2 and 3).

## 4. Mortality Multivariable Analysis

Multivariable regression analysis was done to get the odds ratios (OR) with 95% confidence intervals (CI) with the hospital mortality as the dependent variable. Increasing ∆1 SOFA score (OR = 2.506, 95% CI: 1.681–3.735, *p* < 0.001) and progressive hyperlactatemia (OR = 1.388, 95% CI: 1.015–1.898, *p*=0.04) were significantly associated with hospital mortality after VA-ECMO support for adults with cardiogenic shock. Despite use of central VA-ECMO, haemodialysis, occurrence of ICH, and GI bleeding were significant in the nonsurvivors group, there were not significantly associated with mortality in the regression analysis (Table 7).

## 5. Discussion

We retrospectively analysed our adult patients with cardiogenic shock supported with VA-ECMO to detect the variables associated with hospital mortality in our tertiary care hospital. Our hospital mortality was 56.6%, and most of our patients were males with a mean age of 40.2 ± 14.4 years. This finding is comparable to previous large studies, but our patients were younger than those in other studies.

Schmidt et al. extracted and analysed 3846 patients (mostly males with a median age of 54 years) with VA-ECMO use for refractory cardiogenic shock between the years of 2003 and 2013 from the Extracorporeal Life Support Organization (ELSO) registry and found only 42% survival at hospital discharge [18]. Maxwell et al. studied 8753 patients with ECMO use between the years 1998 and 2009 and found a mean age of 53.9 ± 0.4 years with 51% hospital mortality among all patients and a mean age of 48.9 ± 0.8 years for patients with cardiogenic shock with 64% hospital mortality [19].

El Sibai et al. retrospectively analysed 922 patients with cardiogenic shock on ECMO support from the US Nationwide Emergency Department Sample (NEDS) database and found that most of the patients were males with a mean age of 50.8 years and only 51% of them survived until hospital discharge [20]. Aso et al. analysed 5263 patients with VA-ECMO and found that about 73% of patients were males with a mean age of 64.8 ± 13.7 years and hospital mortality of 73.6% [21].

We studied the variables associated with hospital mortality and found that presence of CKD or development of AKI and use of renal replacement therapy were significant in the nonsurvivors patients. Schmidt et al. found renal failure as a significant variable in the mortality group and used it in creating the survival after venoarterial ECMO (SAVE) score [18]. Wang et al. found association of renal impairment and hospital mortality after CABG and VA-ECMO support and used the serum creatinine as one variable in calculating the REMEMBER score to predict mortality after CABG and VE-ECMO support [22]. However, Aso et al. found that renal impairment was not a significant variable, but the use of renal replacement therapy was significantly associated with mortality [21].

Most of our studied patients had refractory cardiogenic shock due to dilated or ischemic cardiomyopathy and valvular heart disease with about 57.5% of them as postcardiotomy shock. The proportion of adult congenital heart disease, myocarditis, and posttransplantation ECMO was small in our study. There were no significant differences between survivors and nonsurvivors regarding underlying heart disease, but the chronic AF and oral anticoagulation were significantly frequent in the nonsurvivors group. Schmidt et al. detected the better outcome of VA-ECMO for cardiogenic shock of patients with myocarditis or after heart or lung transplantation and the poor outcome of patients with congenital heart diseases, while there were no significant differences for ECMO support of cardiogenic shock due to valvular, ischemic, or other causes [18]. Combes et al. also described the better prognosis of VA-ECMO support for patients with myocarditis [6].

We noticed frequent use of central VA-ECMO in the nonsurvivors group and frequent use of peripheral VA-ECMO in the survivors. This may be related to the frequent cardiac surgeries and related complications like excessive bleeding and associated transfusions. However, central VA-ECMO use was not significant in the multivariate regression analysis model. Our results were going with Mariscalco et al. recent study of 718 adult patients with postcardiotomy shock on VA-ECMO support from 19 cardiac surgical centers. Mariscalco et al. concluded that central VA-ECMO was associated with more hospital mortality, more bleeding, and excess blood products transfusions compared to peripheral VA-ECMO [23]. On the contrary, Djordjevic et al. retrospectively analysed 156 patients with postcardiotomy cardiogenic shock on VA-ECMO support and excluded significant 30 days mortality differences between central and peripheral VA-ECMO groups, but the patients with peripheral ECMO had significantly less mediastinal bleeding, less fresh frozen plasma transfusions, and less mediastinal explorations compared to those with central VA-ECMO support [24].

It is recognized that VA-ECMO decreases the coronary blood flow and increases left ventricular afterload, while IABP could reduce these effects and theoretically promote myocardial recovery and survival [25, 26]. Our results showed absence of significant mortality difference with the concomitant use of IABP and VA-ECMO. This finding is consistent with Schmidt et al.'s study [18] but different from Aso et al.'s [21] study that detected lower mortality with IABP use. Moreover, Wang et al. did not find mortality difference with IABP use either before or after VA-ECMO insertion [22].

The occurrence of gastrointestinal and intracerebral bleeding was significantly frequent in the nonsurvivors group in the univariate analysis but was insignificant in the multivariate regression analysis model. They could be related to the significant coagulopathy and thrombocytopenia or as a part of multiorgan system failure.

Our results showed significantly higher arterial lactate level and greater metabolic acidosis at ECMO initiation in the nonsurvivors group compared to the survivors. Also, the lactate level reached a higher peak with delayed clearance in the nonsurvivors. Hyperlactatemia was significantly associated with mortality in our multivariable regression model (OR = 1.388, 95% CI: 1.015–1.898, *p*=0.04). Schmidt et al. [18] described the significant metabolic acidosis in the nonsurvivors and detected the association of pre-ECMO serum HCO_3_ ≤15 mmol/L and mortality (OR = 0.7, 95% CI: 0.58–0.83, *p* < 0.0001). Moreover, Chen et al. [27] described the greater metabolic acidosis and hyperlactatemia in the nonsurvivors during urgent VA-ECMO insertion for cardiac and noncardiac causes at emergency department and used lactate level to develop the modified SAVE score. The impact of hyperlactatemia on mortality has been reported in adult patients with cardiogenic shock and VA-ECMO support [28, 29].

The use of VA-ECMO especially for refractory cardiogenic shock is a complex bundle of associated measures such as invasive mechanical ventilation and anticoagulation with possible blood products transfusions, cardiac surgery with its related complications, and checking eligibility to heart transplantation or end-of-life decision-making. So we need to score our patients for early decision-making. We used SOFA score for its simplicity and found that initial SOFA assessment was a good predictor of hospital mortality (AUROC: 0.862). Furthermore, increase in SOFA score at the third and fifth days were associated with hospital mortality, while decreasing SOFA score occurred in the survivors. Increased SOFA ≥2 was an independent predictor of hospital mortality (AUROC: 0.958). Schmidt et al. assessed his studied patients with SOFA and APACHE II and III scores at ECMO initiation only and found that the 3 scoring systems were higher in the nonsurvivors, but that study did not follow the patients during VA-ECMO support, and the AUROC was 0.79, 058, and 0.59 for SOFA, APACHE II, and APACHE III, respectively [18].

Chen et al. developed ROC curves to predict 90-day mortality for VA-ECMO patients, the AUROC was 0.65, 0.73, and 0.83 for SOFA, SAVE, and modified SAVE scores at ECMO initiation and that study did not follow-up scoring [27].

The haemodynamics deterioration with cardiogenic shock and resuscitation with ECMO support affects the multiorgan functions that change over time and with the efficiency of resuscitation. So we calculated the SOFA score during the first few days of ECMO support and concluded that increasing SOFA after 48 hours of ECMO support is an independent predictor of hospital mortality.

Ferreira et al. demonstrated the efficacy SOFA scoring of critically ill patients without ECMO support at medicosurgical ICU during the first 96 hours of ICU admission. That study showed the trend of SOFA during the first 48 hours of ICU stay being a sensitive indicator of outcome regardless of the initial SOFA [16].

Jentzer et al. recently studied SOFA scoring of critical patients without ECMO in cardiac ICU and the relation of increased SOFA with increased mortality and the lower long-term survival for survivors with high initial SOFA [30]. Recently, SOFA was used to predict the occurrence of acute cerebral strokes in patients supported with VA-ECMO with a good correlation [31].

Finally, VA-ECMO support is associated with frequent morbidities and high mortality. Using SOFA scoring is a simple way to assess and follow VA-ECMO supported adult patients and help in decision-making.

## 6. Conclusion

The use of VA-ECMO in adult patients with cardiogenic shock is still associated with high mortality. Serial evaluation of those patients with SOFA score during first few days of ECMO support is a good predictor of hospital mortality. Increase in SOFA score after 48 hours and hyperlactataemia are significantly associated with increased hospital mortality.

## Figures and Tables

**Table 1:** Criteria of the sequential organ failure assessment (SOFA) score [15–17].

SOFA score					
The variables	0	1	2	3	4
Respiratory system PaO_2_/FiO_2_ (mmHg)	>400	<400	<300	<200 with respiratory support	<100 with respiratory support
Nervous system Glasgow Coma Scale	15	13-14	10–12	6–9	<6
Cardiovascular system Mean arterial pressure (MAP) or administration of vasopressors required	MAP >70 mmHg	MAP <70 mm/Hg	Dopamine ≤5 *μ*g/kg/min or dobutamine (any dose)	Dopamine >5 *μ*g/kg/min or epinephrine ≤0.1 *μ*g/kg/min or norepinephrine ≤0.1 *μ*g/kg/min	Dopamine >15 *μ*h/kg/min or epinephrine >0.1 *μ*g/kg/min or norepinephrine >0.1 *μ*g/kg/min
Liver Bilirubin (mg/dl) (*μ*mol/L)	<1.2 [<20]	1.2–1.9 [20–32]	2.0–5.9 [33–101]	6.0–11.9 [102–204]	>12.0 [>204]
Coagulation Platelets ×10^3^/ml	>150	<150	<100	<50	<20
Kidneys Creatinine (mg/dl) (*μ*mol/L); urine output	<1.2 [<110]	1.2–1.9 [110–170]	2.0–3.4 [171–299]	3.5–4.9 [300–440] (or urine output <500 ml/day)	>5.0 [>440]; urine output <200 ml/day

**Table 2:** Baseline characteristics of studied patients with VA-ECMO.

Characteristics	All patients (*n* = 106)	Survivors (*n* = 46, 43.4%)	Nonsurvivors (*n* = 60, 56.6%)	*p* value
Age	40.2 ± 14.4	39 ± 10.9	41.1 ± 16.6	0.73
Sex				
Males	74 (69.8)	31 (67.4)	43 (71.7)	0.61
Females	32 (30.2)	15 (32.6)	17 (28.3)	
BMI	26.5 ± 7	26.4 ± 6.6	26.6 ± 7.4	0.82
Diabetes mellitus	20 (18.9)	9 (19.6)	11 (18.3)	0.84
Systemic hypertension	34 (32.1)	14 (30.4)	20 (33.3)	0.75
CKD	21 (19.8)	2 (4.3)	19 (31.7)	0.001
Pre-ECMO AF	36 (34)	9 (19.6)	27 (45)	0.006
Anticoagulants intake	39 (36.8)	10 (21.7)	29 (48.3)	0.005
LVEF%	29.5 ± 13.8	29.2 ± 13.9	29.6 ± 13.9	0.73
Heart disease				
Dilated cardiomyopathy	29 (27.4)	14 (30.4)	15 (25)	0.09
Ischemic cardiomyopathy	26 (24.5)	15 (32.6)	11 (18.3)	
Valvular heart disease	26 (24.5)	7 (15.2)	19 (31.7)	
ACHD	5 (4.7)	1 (2.2)	4 (6.7)	
Others	20 (18.9)	9 (19.6)	11 (18.3)	
Cardiac surgery	61 (57.5)	21 (45.7)	40 (66.7)	0.03
Types of surgery				
CABG	3 (4.9)	2(9.5)	1(2.5)	0.16
Bentall operation	9 (14.7)	3(14.3)	6 (15)	
Valve surgery	22(36.1)	8(38.1)	14(35)	
CABG + valve surgery	10 (16.4)	3(14.3)	7(17.5)	
Heart transplantation	15 (24.6)	5(23.8)	10 (25)	
Pulmonary endarterectomy	2 (3.3)	0	2 (5)	
CPB time (minutes)	231.7 ± 93.5	213.4 ± 83.3	239.9 ± 97.6	0.43
Aortic cross clamping (minutes)	147.1 ± 53	144.3 ± 51.7	148.6 ± 54.6	0.85
IABP	21 (19.8)	12 (26.1)	9 (15)	0.15
Type of ECMO				
Central	46 (43.4)	13 (28.3)	33 (55)	0.006
Peripheral	60 (56.6)	33 (71.7)	27 (45)	
ECMO days	9.7 ± 7.2	10.1 ± 6.6	9.5 ± 7.7	0.21
Upgrading to LVAD	15 (14.2)	9(19.6)	6(10)	0.03
ICU days	16.5 (10–32)	20 (14–57)	14 (5.5–30.5)	0.002
Post-ICU days	16 (10–25)	16 (10–25)	10 (4–16)	0.23
Ventilator days	10.5 (6–26)	9 (8–26)	13.5 (5–25.5)	0.95
AKI	73 (68.9)	21 (45.7)	52 (86.7)	<0.001
Haemodialysis	50 (47.2)	9 (19.6)	41 (68.3)	<0.001
Gastrointestinal bleeding	24(22.6)	6 (13)	18 (30)	0.039
Ischemic stroke	13 (12.3)	3 (6.5)	10 (16.7)	0.11
Intracerebral haemorrhage	13 (12.3)	1 (2.2)	12 (20)	0.006

Data are presented mean ± SD, median (IQR), or *N* (%).

**Table 3:** Laboratory data at ECMO insertion.

Characteristics	All patients	Survivors	Nonsurvivors	*p* value
aPTT (seconds)	51.3 ± 26.1	44.4 ± 12.1	56.7 ± 32.2	0.06
PTT ratio	1.5 ± 0.8	1.3 ± 0.4	1.6 ± 0.9	0.14
INR	1.7 ± 0.5	1.7 ± 0.6	1.7 ± 0.4	0.75
Fibrinogen (g/L)	3.3 ± 1.3	3.5 ± 1.4	3 ± 1.1	0.24
Platelet count (10^9^/L)	164.5 ± 90.8	177.9 ± 85.4	154.2 ± 94.2	0.11
Haemoglobin (gm/L)	114.5 ± 19.8	114.4 ± 17.4	114.6 ± 21.6	0.78
Base excess (mmol/L)	−9 ± 3.7	−7.3 ± 3.6	−10.4 ± 3.1	<0.001
Serum lactate (mmol/L)	6 ± 2.3	4.4 ± 1.5	7.2 ± 2	<0.001
Serum creatinine (*μ*mol/L)	112.9 ± 64.9	96.4 ± 46	125.5 ± 74.2	0.018
Serum bilirubin (*μ*mol/L)	30.3 (18.9–58.9)	28.6 (15.7–58.7)	37 (22–61.7)	0.09

Data are presented mean ± SD, median (IQR), or *N* (%).

**Table 4:** Laboratory data during ECMO support.

Characteristics	All patients	Survivors	Nonsurvivors	*p* value
aPTT (seconds)	58.8 ± 13.9	60 ± 9	57.8 ± 16.8	0.02
PTT ratio	1.7 ± 0.4	1.8 ± 0.3	1.7 ± 0.5	0.01
INR	1.8 ± 0.9	1.6 ± 0.4	1.9 ± 1.1	0.09
Fibrinogen (g/L)	3.3 ± 1.5	3.2 ± 1.1	3.3 ± 1.8	0.82
Platelet count (10^9^/L)	104 ± 67	135.1 ± 55.3	80.2 ± 65.7	<0.001
Peak lactate level(mmol/L)	14.2 ± 4.3	11 ± 3	16.7 ± 3.3	<0.001
Lactate at 24 hours	5.4 ± 5.2	2.2 ± 0.9	7.9 ± 5.7	<0.001
Serum creatinine (*μ*mol/L)	139.2 ± 66	122.4 ± 57.6	151.9 ± 69.5	0.017
Serum bilirubin (*μ*mol/L)	84.1 (48.7–183)	78.3 (34.9–172)	84.3 (60.3–267)	0.03
Serum albumin (g/L)	32.8 ± 5	35.2 ± 3.6	31 ± 5.2	0.01

Data are presented mean ± SD, median (IQR), or *N* (%).

**Table 5:** SOFA scoring of studied VA-ECMO patients.

SOFA scores	All patients	Survivors	Nonsurvivors	*p* value
SOFA day 1	13.6 ± 3.7	10.9 ± 2.8	15.6 ± 2.9	<0.001
SOFA day 3	14.7 ± 5.8	8.8 ± 2.6	19.2 ± 2.5	<0.001
SOFA day 5	14 ± 6.6	7.6 ± 2.2	19.8 ± 2.5	<0.001
∆1 SOFA (day 3-1)	2 (−2–4)	−2 (−3 to −1)	4 (3–5)	<0.001
∆2 SOFA (day 5-1)	1 (−4–4.5)	−4 (−5 to −2)	4 (3–6)	<0.001

Data are presented mean ± SD or median (IQR).

**Table 6:** The validity measures of SOFA scoring of studied VA-ECMO patients.

SOFA scores	AUROC	95% CI	Cut-off	Sensitivity (%)	Specificity (%)	PPV (%)	NPV (%)	Accuracy (%)
SOFA day 1	0.862	0.79–0.93	≥13	85.0	73.9	81.0	79.1	80.2
SOFA day 3	0.995	0.986–1.0	≥13	100.0	91.3	93.8	100	96.2
SOFA day 5	0.994	0.982–1.0	≥15	98.0	100.0	100	97.9	99.0
∆1 SOFA (3-1)	0.958	0.913–1.0	≥2	95.0	93.5	95.0	93.5	94.3
∆2 SOFA (5-1)	0.958	0.909–1.0	≥2	90.0	97.8	97.8	90.0	94.8

AUROC: area under ROC; CI: confidence interval; PPV: positive predictive value; NPV: negative predictive value.

**Table 7:** Multivariable regression analysis of hospital mortality of patients with cardiogenic shock supported with VA-ECMO.

Studied variables	*p* value	OR	95% CI for OR
Central VA-ECMO	0.248	3.102	0.455–21.154
Hyperlactatemia	0.040	1.388	1.015–1.898
Haemodialysis	0.712	1.614	0.127–20.473
∆1 SOFA (3-1)	<0.001	2.506	1.681–3.735
GI bleeding	0.917	0.891	0.102–7.815
ICH	0.424	14.585	0.020–204.51

## Data Availability

The data used to support the findings of the study are available from the corresponding author on request.

## Abbreviations

ACHD: Adult congenital heart disease
ACT: Activated clotting time
AKI: Acute kidney injury
AF: Atrial fibrillation
APACHE: Acute physiology, age, and chronic health evaluation
aPTT: Activated partial thromboplastin time
BMI: Body mass index
CABG: Coronary artery bypass grafting
CKD: Chronic kidney disease
CPB: Cardiopulmonary bypass
DM: Diabetes mellitus
EF: Ejection fraction
GI bleeding: Gastrointestinal bleeding
IABP: Intra-aortic balloon pump
ICH: Intracerebral haemorrhage
INR: International normalized ratio
LVAD: Left ventricular assist device
ROC: Receiver operating characteristic
SOFA: Sequential organ failure assessment
OR: Odds ratio
VA-ECMO: Venoarterial extracorporeal membrane oxygenation.

## Additional Points

Our work was a single center retrospective study with a relatively limited number of patients. It needs further larger prospective validation studies.
